# The symbiotic magnetic-sensing hypothesis: do *Magnetotactic Bacteria* underlie the magnetic sensing capability of animals?

**DOI:** 10.1186/s40462-017-0113-1

**Published:** 2017-10-23

**Authors:** Eviatar Natan, Yoni Vortman

**Affiliations:** 1 The Aleph Lab, Oxford, OX2 8NU UK; 2 Hula Research Center, Department of Animal Sciences, Tel-Hai College, Kiryat Shmona, Israel

**Keywords:** Movement ecology, Magnetoreception based navigation, Magnetotactic bacteria (MTB), Bacteria-host relationship, Lacrimal glands

## Abstract

The ability to sense Earth’s magnetic field has evolved in various taxa. However, despite great efforts to find the ‘*magnetic-sensor*’ in vertebrates, the results of these scientific efforts remain inconclusive. A few decades ago, it was found that bacteria, known as magnetotactic bacteria (MTB), can move along a magnetic field using nanometric chain-like structures. Still, it is not fully clear why these bacteria evolved to have this capacity. Thus, while for MTB the ‘*magnetic-sensor*’ is known but the adaptive value is still under debate, for metazoa it is the other way around.

In the absence of convincing evidence for any ‘*magnetic-sensor*’ in metazoan species sensitive to Earth’s magnetic field, we hypothesize that a mutualism between these species and MTB provides one. In this relationship the host benefits from a magnetotactic capacity, while the bacteria benefit a hosting environment and dispersal. We provide support for this hypothesis using existing literature, demonstrating that by placing the MTB as the ‘*magnetic-sensor*’, previously contradictory results are now in agreement. We also propose plausible mechanisms and ways to test the hypothesis. If proven correct, this hypothesis would shed light on the forces driving both animal and bacteria magnetotactic abilities.

## Background

### Magnetoreception-based navigation

The geomagnetic field is an omnipresent feature of Earth. It is therefore not surprising that various organisms, including invertebrates, vertebrates and bacteria, use magnetotactic abilities for orientation and navigation [1–6]. Magnetoreception has been of significant scientific interest [1, 5, 7–9] both for its navigation-based capacity as well for other uses [5, 10]. Intensive work has demonstrated the existence of magnetic sensing and its possible function in animal navigation [11–13]. Earth’s magnetic field is rather weak, with 60–65 μT at the poles, and 25–30 μT near the equator [14]. To put this in perspective, a standard fridge magnet produces a magnetic force that is ~200 times stronger. Thus, sensing a magnetic field as weak as the Earth’s is challenging. Most of magnetoreceptive animals sense the magnetic field’s inclination angle, meaning the magnetic field lines, rather than the magnetic field polarity, i.e. north and south [1, 8, 13]. This provides the animal with a proxy for its latitude, an essential measure for wandering or long-distance migrating animals. The ability of animals to sense the magnetic field’s inclination angle has been well documented for over 40 years [1] and has been reported in various taxa across the animal kingdom including fish [15], insects [16] and apparently all tetrapods except mammals, e.g. reptiles [8] and birds [1];reviewed in ref. [11]. However, the sensor and sensory mechanism behind this remain an enigma and are widely debated. In vertebrates, for example, it has been suggested that the *‘magnetic-sensor’* or sensing organ is located in the ethmoid region of the head, between the eyes orbits and the naris [17–19].

To date, two not necessarily mutually exclusive hypotheses to explain magnetic-sensing in animals have been proposed [20]: (*i*) “*radical-pair*” based magnetoreception [21] and (*ii*) magnetite-based magnetoreception [2]. The “*radical-pair*” hypothesis suggests that following a short wavelength excitation, a specific molecule that contains two unpaired electrons, such as the cryptochrome protein, could serve as the sensor for Earth’s magnetic field. The magnetite-based magnetoreception hypothesis suggests that biogenic magnetite crystals serve as Earth’s magnetic field sensors. Support and criticism for both hypotheses are summarized in Table 1.

**Table 1:** Support and criticism for the “radical-pair” and “magnetite-based” magnetoreception hypotheses

	Support	Criticism
Radical–Pair	Experiments showing that birds can only sense the magnetic field under illumination with relative short wavelength, as opposed to longer wavelengths [41]	The effect of the magnetic field on the spin-state of the molecule has not been demonstrated, either in vitro or in vivo, under the Earth’s weak magnetic field, but only under a field orders of magnitude stronger [20, 47]
		The activation mechanism is missing, meaning how the signal transduces to initiate a neural response.
Magnetite-Based	Magnetite crystals have been detected in magnetic sensing fish, reptiles and birds [18, 28, 48]	The magnetites found in some magnetic-sensing animals are not associated to the animals’ neuronal, or other tissue, but rather located in macrophages [30] or as contaminants [32]
	Magnetotactic-bacteria (MTB) can act upon the field via similar magnetite crystals [2, 22]	No one has seen magnetite crystals serving as a ‘*magnetic-sensor*’ except in bacteria [20] The activation mechanism is missing, meaning how the signal transduces to initiate a neural response.

### Magnetotactic bacteria

In contrast to all other organisms, an unequivocal demonstration of the use of the geomagnetic field’s inclination angle for orientation was found in magnetotactic bacteria (MTB). Magnetotactic bacteria (MTB) are a diverse group of aquatic prokaryotes, ubiquitously found in both fresh and marine sediment habitats. MTB show magnetotaxis - the ability to align with a magnetic field using specialized intracellular organelles called magnetosomes [22]. In most MTB, magnetosomes comprise chain-like, nanometer-sized crystals (typically between 35 and 120 nm) of magnetic iron minerals [22]. It has been suggested that the magnetotaxis redirects MTB towards an anaerobic environment [23]. In aquatic environments, there are opposing gradients of oxygen and sulfide from the surface to the bottom of the sediment, which create an oxi-anoxic transition zone (OATZ). Most MTB prefer to reside at, or close to, the OATZ. While recent experiments support the general assumption that MTB use the magnetic field to vertically locate the OATZ [24, 25] there are a few unresolved issues with this model. For example, MTB recognize and move along the oxygen gradient even if the magnetic cue directs them against it [23]. In addition, some MTB produce a large number of magnetosomes, far greater than would be needed to align along Earth’s magnetic field [26]. Finally, MTB have been found at, or near the equator, where magnetotaxis has no advantage in directing vertical movement, as the inclination angle is ~0° [27]. These confounding observations elicited the formulation of alternative hypotheses as to the possible advantages gained by MTB through the production of magnetosomes, yet none of these hypotheses have been proven conclusive [23]. Although the adaptive value of MTB’s magnetotaxis is still under some debate, MTB’s ability to act upon the magnetic field inclination angle is well established.

In contrast to MTB, no one has directly observed magnetite crystals serving as a ‘*magnetic-sensor*’ in animals [20]. This is despite repeated reported observations of magnetite crystals, in various taxa [1, 2, 18].

## Main text

### The symbiotic magnetic-sensing hypothesis and suggested mechanisms

**Fig. 1:**
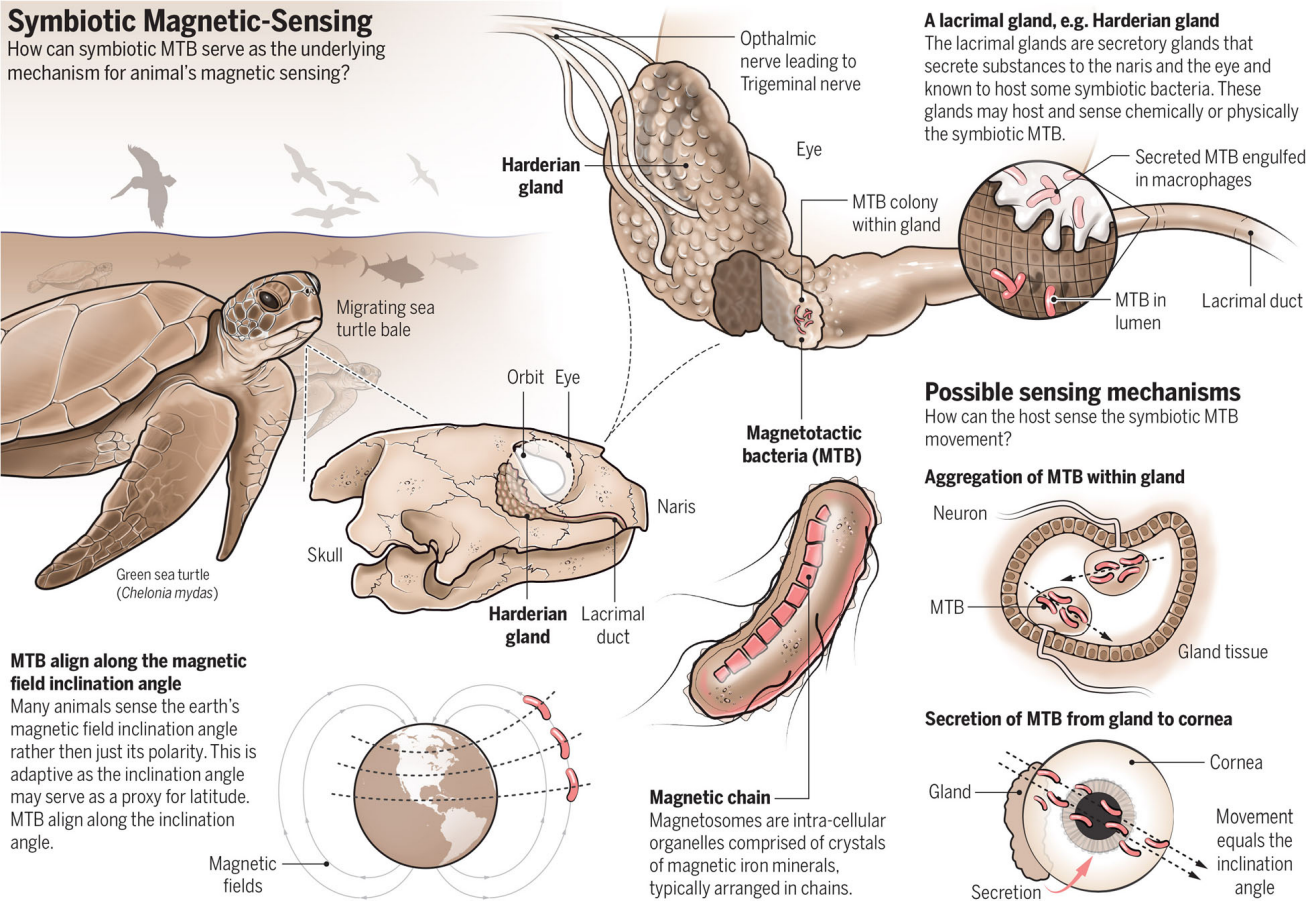
Visual abstract of the symbiotic magnetotactic hypothesis

Here, we suggest that MTB serve a symbiotic function, providing a ‘*magnetic-sensor*’ for the host. We further suggest that the lacrimal glands of vertebrates are potential habitats for symbiotic MTB. The hypothesis is summarized in Fig. 1.

Our hypothesis is derived from the magnetite-based magnetoreception hypothesis. As mentioned above, magnetite crystals have been found in a large number of organisms [2, 20, 28], including birds [29], and have been described as being “strikingly similar to the MTB crystals” [2]. However, magnetite crystals have not convincingly been located within bird tissues or neuron cells [30–32] but instead appear to be extracellular contamination [32] or within macrophages [30]. Interestingly, it is known that macrophages engulf commensal bacteria, so it is possible that these crystals may have originated from MTB.

The magnetite-based magnetoreception hypothesis requires magnetite to be organized in a chain-like manner [33], similar to that seen in MTB, as the crystals individually are too small to contribute to effective magnetoreception. The majority of magnetic-sensing animals use an inclination compass, as MTB do. This similarity may be because MTB underlie the animal’s inclination sensing capacity; alternatively, the capacity may have evolved independently in both kingdoms. In addition, the ubiquity of MTB means that the host is constantly exposed to them.

Previous studies have suggested that the magnetic sensing organ is located in the ethmoid region of the head [20]. Within the eye orbit, there are several glands such as the Harderian and other lacrimal glands [34]. The primary function of these glands is bathing and lubricating the eye, but they also serve other functions including photoreception, immunocompetence and habitats for endosymbiotic bacteria [34, 35]. The lacrimal glands also secret substances to the naris or the eye, such as hormones, porypherins and symbiotic bacteria [34, 35].

Studies have shown that a complex nerve system, whose function is unknown, runs through the glands and is associated with the ophthalmic nerve, e.g. ref. [36]. Sectioning of the ophthalmic nerve impairs birds’ magnetic sensing [19], while other works support a role for the visual system [37, 38]. The Harderian gland surrounds the ophthalmic nerve, thus experiments sectioning that nerve have impaired the nerves that are connected to the gland.

There are several plausible mechanisms by which the MTB and host may communicate, enabling the host to sense the magnetic field. One possible mechanism is by the bacteria moving and accumulating at a specific location on the gland, allowing cell-to-cell communication. Whether the MTB move passively or actively in response to the magnetic field, their movement and/or accumulation could be detected by the host (Fig. 1). Interestingly, most MTB show exceptionally high number of proteins that are predicted to have functions in chemotaxis, sometimes order of magnitude higher than other types of bacteria [39]. These secretions may excite a specific nerve connected to the ophthalmic nerve, passing on information to the host about the Earth’s magnetic field. This type of communication between commensal bacteria and the host nervous system has been shown in various animal systems [40].

A different mechanism by which the host could detect the MTB movement could be through the host’s visual system. For example, secreted bacteria from the lacrimal glands may move along the cornea. This movement could be perceived by the visual system similarly to the way in which humans perceive blood droplets movement along the cornea. It has been shown that birds sense the magnetic field under illumination of a relative short (443-550 nm), but not long (630 nm), wavelength [41], which means that the detection of small objects such as bacteria is a valid possibility. It should be noted that these experiments have been proposed to support the “*radical–pair*” hypothesis [41]. Collectively, the “*radical-pair*” supporting experiments are not in conflict with the possibility of MTB being the underline mechanism of animals’ magnetic sensing. In fact, MTB magnetic orientation is also affected by light: green light decreases the translation velocity whereas red light increases it, in comparison to blue and white light [42].

The results from experiments in which an animal’s magnetic sensing has been manipulated are consistent with both proposed mechanisms [19, 37, 38, 41] as sectioning of the ophthalmic nerve may alter sensing or secretion of bacteria from the gland. Similarly, lack of illumination may affect magnetic sensing by making bacteria less detectable or reducing the secretory response within the gland [34].

Handling and isolating anaerobe MTB requires dedicated methods [22]. Naturally, previous studies aiming to detect magnetite in metazoan used methods that were not suitable for identifying MTB, yet recent methodological progress (e.g. magnetoscope) should aid in bridging this gap [32, 43]. Recent progress in molecular methods has greatly increased the knowledge of genes that regulate magnetosome formation [39, 44]. These genes could potentially serve to detect symbiotic MTB. However, due to their polyphyletic nature and great variation [45], to date there is no general primer set which would enable the detection of all MTB species.

## Conclusion

It was well established that animals’ behavior can be manipulated by microorganisms. For example, recent evidence show that the gut microbiota integrate into the gut–brain axis interact to change brain function [40], or the multitude effect of parasites on the hosts’ personality and behavior [46]. We predict that similar mechanisms between the host and the microorganism exist to generate the magnetic sensing capability in birds and possibility other animals.

Previously, the presence of magnetite in many animals and fossils, led Kirschvink et al. to hypothesize that magnetite crystals may have been intra-cellularly incorporated, similarly to the endosymbiosis incorporation of mitochondria billions of years ago [2]. Here, we suggest that extant animal magnetotactic abilities are *still* endosymbiont, meaning the bacteria reside side by side to the eukaryote cells.

The proposed symbiotic magnetic sensing hypothesis can be proved or refuted experimentally using an approach similar to Koch’s Postulates. If proved correct, this hypothesis will shed light on the ecological and evolutionary forces driving, maintaining, and shaping magnetic-sensing abilities for both bacteria and animals, solving a long-lasting scientific mystery.
